# Evaluation of Adipose Tissue Zinc-Alpha 2-Glycoprotein Gene Expression and Its Relationship with Metabolic Status and Bariatric Surgery Outcomes in Patients with Class III Obesity

**DOI:** 10.3390/biomedicines10071502

**Published:** 2022-06-25

**Authors:** José Ignacio Martínez-Montoro, Luis Ocaña-Wilhelmi, Rocío Soler-Humanes, Hanieh Motahari-Rad, Andrés González-Jiménez, José Rivas-Becerra, Alba Rodríguez-Muñoz, Francisco J. Moreno-Ruiz, Mónica Tomé, Jorge Rodríguez-Capitán, Eduardo García-Fuentes, Francisco J. Tinahones, Lourdes Garrido-Sánchez, Mora Murri

**Affiliations:** 1 Clinical Management Unit (UGC) of Endocrinology and Nutrition, Virgen de la Victoria University Hospital, 29010 Malaga, Spain; joseimartinezmontoro@gmail.com (J.I.M.-M.); lourgarrido@gmail.com (L.G.-S.); moramurri@gmail.com (M.M.); 2 Instituto de Investigación Biomédica de Malaga (IBIMA), 29010 Malaga, Spain; doctoratome@gmail.com (M.T.); capijorge@hotmail.com (J.R.-C.); 3 Faculty of Medicine, University of Malaga, 29010 Malaga, Spain; albarodriguezmunoz9@gmail.com; 4 Clinical Management Unit (UGC) of General and Digestive Surgery, Virgen de la Victoria University Hospital, 29010 Malaga, Spain; luisowilhelmi@hotmail.com (L.O.-W.); rocioshumanes@hotmail.com (R.S.-H.); 5 Department of Molecular Genetics, Faculty of Biological Sciences, Tarbiat Modares University, Tehran 14117-13116, Iran; haniemotahary@gmail.com; 6 Common Support Structures (ECAI) Bioinformática, Instituto de Investigación Biomédica de Malaga (IBIMA), 29010 Malaga, Spain; bioinformatica@ibima.eu; 7 Department of General, Digestive and Transplant Surgery, Regional University Hospital of Malaga, 29010 Malaga, Spain; doctopep@hotmail.com (J.R.-B.); javier.morenoruiz@gmail.com (F.J.M.-R.); 8 Department of Endocrinology and Nutrition, Regional University Hospital of Malaga, 29010 Malaga, Spain; 9 Clinical Management Unit (UGC) of Heart, Virgen de la Victoria University Hospital, 29010 Malaga, Spain; 10 Centro de Investigación Biomédica en Red de Enfermedades Cardiovasculares (CIBEROBN), Instituto de Salud Carlos III, 28029 Madrid, Spain; 11 Department of Gastroenterology, Virgen de la Victoria University Hospital, 29010 Malaga, Spain; 12 Centro de Investigación Biomédica en Red de la Fisiopatología de la Obesidad y Nutrición (CIBEROBN), Instituto de Salud Carlos III, 28029 Madrid, Spain

**Keywords:** adipose tissue, zinc-α2 glycoprotein, gene expression, metabolic syndrome, bariatric surgery, morbid obesity

## Abstract

Zinc-α2 glycoprotein (ZAG) is an adipokine involved in adipocyte metabolism with potential implications in the pathogenesis of metabolic disorders. Our aim was to evaluate the relationship between visceral (VAT) and subcutaneous adipose tissue (SAT) *ZAG* expression and metabolic parameters in patients with class III obesity, along with the impact of basal ZAG expression on short- and medium-term outcomes related to bariatric surgery. 41 patients with class III obesity who underwent bariatric surgery were included in this study. *ZAG* gene expression was quantified in SAT and VAT. Patients were classified into two groups according to SAT and VAT ZAG percentile. Anthropometric and biochemical variables were obtained before and 15 days, 45 days, and 1 year after surgery. The lower basal SAT *ZAG* expression percentile was associated with higher weight and waist circumference, while the lower basal VAT *ZAG* expression percentile was associated with higher weight, waist circumference, insulin, insulin resistance, and the presence of metabolic syndrome. Basal SAT *ZAG* expression was inversely related to weight loss at 45 days after surgery, whereas no associations were found between basal VAT *ZAG* expression and weight loss after surgery. Additionally, a negative association was observed between basal SAT and VAT *ZAG* expression and the decrease of gamma-glutamyl transferase after bariatric surgery. Therefore, lower SAT and VAT *ZAG* expression levels were associated with an adverse metabolic profile. However, this fact did not seem to confer worse bariatric surgery-related outcomes. Further research is needed to assess the clinical significance of the role of *ZAG* expression levels in the dynamics of hepatic enzymes after bariatric surgery.

## 1. Introduction

Current epidemiological data show a dramatic increase in worldwide prevalence of overweight and obesity over the last few decades, with more than 2.1 billion people affected globally by these conditions [1]. Obesity is associated with a number of comorbidities such as cardiovascular disease, dyslipidemia, insulin resistance, type 2 diabetes mellitus (T2DM), immune dysfunction, and certain types of cancer [2]. Importantly, this disease constitutes a major health problem as it is associated with an increased risk of morbidity and mortality, causing 3.4 million deaths and 3.9% of years of life lost worldwide [3].

Essentially, the cause of obesity and overweight is an energy imbalance between calories consumed and calories expended. Thus, lifestyle modifications are the cornerstone in the treatment of this disease. However, bariatric surgery (BS) is probably the only available option to achieve long-term maintenance of substantial weight loss and improve obesity-related comorbidities in the most extreme form of obesity, named morbid obesity [4].

In recent years, adipose tissue has been recognized as a major endocrine organ which plays a key role in energy homeostasis. There are two main types of adipose tissue: white adipose tissue (WAT) and brown adipose tissue (BAT) [5]. WAT, in turn, can be classified in subcutaneous WAT (SAT) and visceral WAT (VAT) [5]. In the presence of excess energy supply, WAT expands as a result of cellular hypertrophy and hyperplasia [5,6]. Of note, hypertrophic adipocytes become frequently dysregulated, which leads to an altered release of adipokines [5]. With regard to this fact, zinc-α2-glycoprotein (ZAG) has emerged as a novel adipokine, which may have a role in the adipocyte metabolism as a modulator of lipid mobilization and adipokine production [7]. ZAG expression has been found to be downregulated in subjects with obesity and this fact could contribute to an impaired lipid mobilization and increased fat accumulation in SAT and VAT in this population [8]. 

On the other hand, there is a well-known link between excess adiposity observed in obesity and insulin resistance (IR) and altered glucose metabolism [9]. In this line, SAT ZAG has been shown to correlate positively with insulin sensitivity in obesity, while circulating ZAG levels are lower in patients with impaired glucose tolerance, T2DM, and polycystic ovary syndrome [10]. Moreover, decreased circulating ZAG levels in serum have been associated with the metabolic syndrome and central obesity and may constitute a useful biomarker for the diagnosis of this disease [11].

Previously, we studied ZAG expression levels in patients with class III obesity [12]. Our analyses showed that VAT *ZAG* expression levels were higher in patients with morbid obesity and low IR compared with those presenting high IR, and VAT *ZAG* expression levels were independently related to insulin, homeostatic model assessment of insulin resistance (HOMA-IR), and adiponectin [12]. On the other hand, contradictory results have been reported regarding *ZAG* levels after BS [13,14]. However, to our knowledge, there are no available studies assessing the influence of *ZAG* expression on BS outcomes.

Hence, in the current work, our aim was to assess the relation of SAT and VAT *ZAG* gene expression levels with basal metabolic characteristics of patients with class III obesity and with short- and medium-term BS-related outcomes, evaluating the potential role of SAT and VAT *ZAG* as predictive markers of BS-related out-comes. The potential implication of the findings of this study may also help to attain a better comprehension of the role of this adipokine in this severe form of obesity.

## 2. Materials and Methods

### 2.1. Subjects 

A total of 41 patients with class III obesity (body mass index-BMI 54 ± 7 kg/m^2^), undergoing BS (biliopancreatic diversion of Scopinaro or sleeve gastrectomy), were included in this study. 

Clinical, anthropometric, and biochemical data were obtained before and 15 days, 45 days, and 1 year after surgery. Exclusion criteria comprised the presence of T2DM, established cardiovascular disease, arthritis, or acute inflammatory disease, infectious disease, or the administration of drugs at the time of the inclusion that could alter the lipid profile or other biochemical parameters. The study was conducted in accordance with the Declaration of Helsinki and was approved by the Research Ethics Committee of Malaga (CEI_PI-0194-2017). Informed consent was obtained from all patients participating in this study. 

### 2.2. Laboratory Measurements 

Blood samples were collected after 12 h of fasting. Serum samples were separated and immediately frozen at −80 °C. Serum biochemical parameters were measured in duplicate. Serum glucose, triglycerides, cholesterol, and high-density lipoprotein (HDL) cholesterol levels were measured by standard enzymatic methods (Randox Laboratories Ltd., Antrium, UK). C-reactive protein (CRP), adiponectin, and leptin levels were measured by enzyme immunoassay (ELISA) kits (BLK Diagnostics, Badalona, Spain, DRG Diagnostics, Marburg, Germany and Mediagnost, Reutlingen, Germany, respectively) [12,15]. Insulin was determined by immunoradiometric assay (BioSource International, Camarillo, CA, USA), showing a 0.3% cross-reaction with proinsulin. HOMA-IR was calculated using the following equation: HOMA-IR = fasting insulin (µIU/mL) × fasting glucose (mol/L)/22.5.

### 2.3. Visceral and Subcutaneous Adipose Tissue mRNA

We analyzed basal *ZAG* gene expression levels in VAT and SAT, using the samples obtained during BS. The samples were washed in physiological saline and immediately frozen in liquid nitrogen and stored at −80 °C until analysis. Total RNA was extracted using RNeasy lipid tissue midi kit (QIAGEN Science, Hilden, Germany), and treated with 55 U RNase-free deoxyribonuclease (QIAGEN Science, Hilden, Germany) in accordance with the manufacturer’s instructions. The purity of the RNA was determined by absorbance at 260/absorbance at 280 ratio on the Nanodrop ND-1000 spectrophotometer (Thermo Fisher Scientific Inc. Waltham, MA, USA). Total RNA was reverse transcribed to cDNA by the utilization of a high-capacity cDNA reverse transcription kit (Applied Biosystems, Foster City, CA, USA). RT-qPCR reactions were performed using TaqMan^®^ Gene Expression Assays (Applied Biosystems, Foster City, CA, USA) on an ABI 7500 Fast Real-Time PCR System (Applied Biosystems, Foster City, CA, USA). Additionally, we analyzed the relative basal mRNA expression levels of *ZAG* (Hs00426651_m1, RefSeq. NM_00185.3). mRNA transcripts were normalized to the expression of cyclophilin A (*PPIA*) (4326316E, RefSeq. NM_021130.3). Quantification of transcript level by RT-PCR was done by the use of relative Ct method (2^−ΔΔCt^).

### 2.4. Statistical Analysis

Data were expressed as mean values ± SD, unless otherwise stated. Clinical and biochemical data were analyzed by mean comparison (*t*-student test or Mann–Whitney U, depending on the normal distribution). Analysis of variance when the same measurement was made several times on each case was performed by generalized linear model (GLM) repeated measures. After testing the normal distribution of the continuous variables by the Shapiro–Wilk test, we applied logarithmic transformation as needed to ensure normality of skewed variables in GLM repeated measures analysis. Relationships between *ZAG* gene expression levels and clinical/biochemical variables were analyzed by Spearman’s correlation test. Linear regressions were used to determine the association between variables. The statistical analysis was performed with SPSS (Version 26.0 for Windows; SPSS, Chicago, IL, USA). The level of significance was set at *p* < 0.05. 

## 3. Results

### 3.1. Basal Characteristics of the Study Population

**Figure 1:**
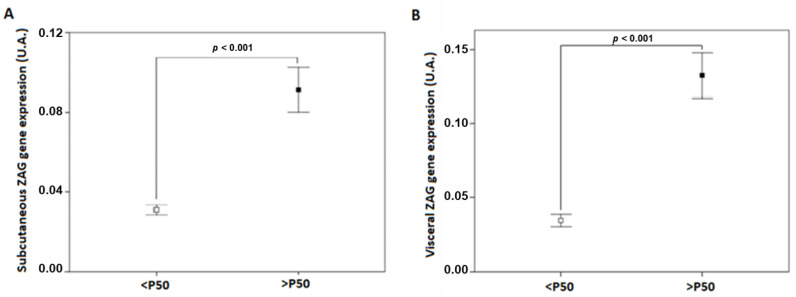
*ZAG* gene expression levels in SAT (**A**) and VAT (**B**) adipose tissue of patients with class III obesity. P50, the 50th percentile of ZAG; SAT, subcutaneous adipose tissue; VAT, visceral adipose tissue; ZAG, zinc-α2 glycoprotein.

Study participants were stratified by the 50th percentile of SAT and VAT *ZAG* gene expression levels (Figure 1). Clinical and biochemical characteristics of the study participants are shown in Table 1. Significant differences were observed between SAT ZAG groups regarding waist circumference, weight, and some other components of the metabolic syndrome. Similarly, VAT ZAG groups showed differences in waist circumference and weight, and some additional characteristics concerning the metabolic syndrome, such as insulin and HOMA-IR. 

### 3.2. Relationship between Basal SAT and VAT ZAG Gene Expression Levels and Clinical Variables

**Figure 2:**
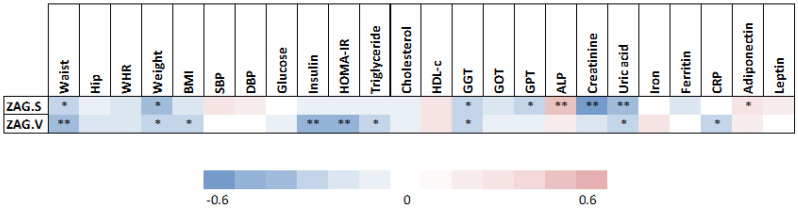
Correlations between clinical and metabolic parameters and adipose tissue *ZAG* levels. Abbreviations: ALP, alkaline phosphatase; BMI, body mass index; CRP, c reactive protein; DBP, diastolic blood pressure; GGT, gamma-glutamyl transferase; GOT, glutamic oxaloacetic transaminase; GPT, glutamic pyruvic transaminase; HDL-c, high-density lipoprotein cholesterol; HOMA-IR, homeostasis model assessment of insulin resistance; SBP, systolic blood pressure; WHR, waist to hip ratio; ZAG.S; subcutaneous adipose tissue zinc-α2 glycoprotein; ZAG.V; visceral adipose tissue zinc-α2 glycoprotein. Statistical significance is expressed as: * *p* < 0.05; ** *p* < 0.01.

A significant negative correlation was found between basal SAT *ZAG* gene expression and waist circumference, weight, gamma-glutamyl transferase (GGT), glutamic pyruvic transaminase (GPT), creatinine and uric acid; whereas a significant positive correlation was observed between basal SAT *ZAG* gene expression and alkaline phosphatase (ALP) and adiponectin (Figure 2). On the other hand, a significant negative correlation was observed between basal VAT *ZAG* gene expression and waist circumference, weight, BMI, insulin, HOMA-IR, triglycerides, GGT, uric acid and C- reactive protein (CRP) (Figure 2).

To further analyze the impact of the distinct clinical parameters, we performed different linear regression models, including those parameters that significantly correlated with SAT and VAT *ZAG* gene expression levels. We included the gene expression levels of SAT *ZAG* as dependent variable and sex, waist circumference, weight, SBP, GGT, ALP, creatinine, and ferritin levels as independent variables. The first regression model (R^2^ = 0.270, F = 13, *p* = 0.001) suggested that 27% of the gene expression levels of SAT *ZAG* could be explained by a negative effect of creatinine (ß = −0.520, *p* = 0.001). The second regression model, which included the gene expression levels of VAT *ZAG* as dependent variable and the presence of metabolic syndrome, waist circumference, weight, insulin, HOMA-IR, and iron levels as independent variables (R^2^ = 0.422, F = 9, *p* < 0.001), demonstrated that 42% of VAT *ZAG* gene expression levels could be explained by a negative effect of waist circumference (ß = −0.276, *p* = 0.049), metabolic syndrome (ß = −0.306, *p* = 0.022), and insulin levels (ß = −0.336, *p* = 0.018).

On the other hand, regression models suggested that SAT *ZAG* gene expression levels could explain 10% of the variation of waist circumference (R^2^ = 0.106, ß = −0.326, *p* = 0.049), 27% of creatinine levels (R^2^ = 0.270, ß = −0.520, *p* = 0.001), and 12% of uric acid levels (R^2^ = 0.121, ß = −0.348, *p* = 0.035). Moreover, regression models suggested that variations of waist circumference, weight, and BMI (21%, 14%, and 12%, respectively) could be explained by VAT *ZAG* gene expression levels (R^2^ = 0.207, ß = −0.455, *p* = 0.003; R^2^ = 0.136, ß = −0.369, *p* = 0.018; R^2^ = 0.117, ß = −0.342, *p* = 0.029, respectively). Furthermore, regression models suggested that the variation of 25% of insulin levels and 23% of HOMA-IR could be explained by VAT *ZAG* gene expression levels (R^2^ = 0.247, ß = −0.497, *p* = 0.001; R^2^ = 0.232, ß = −0.482, *p* = 0.001, respectively). In addition, 11% of the variation of GGT levels could be explained by VAT *ZAG* gene expression levels (R^2^ = 0.107, F = 4.7, ß = −0.327, *p* = 0.037).

### 3.3. Changes in the Different Clinical and Analytical Parameters after Bariatric Surgery

Table 2 and Table 3 display the changes in the different clinical and analytical parameters before BS (Basal) and after BS (15 days, 45 days, and 1 year) according to the lower and upper 50th percentile of basal SAT and VAT *ZAG* gene expression, respectively. Briefly, significant body weight reductions, along with BMI reductions, were observed as early as 15 days after BS in both percentile groups of basal SAT and VAT *ZAG* gene expression and continued gradually decreasing until 1 year after the intervention. Similar dynamics were found in other parameters such as insulin, HOMA-IR, and leptin. Systolic blood pressure was significantly reduced 15 days after BS in both the lower and the upper percentile of basal SAT and VAT *ZAG* gene expression and continued until 1 year after BS, whereas similar changes were found in diastolic blood pressure in subjects within the lower percentile of basal SAT and VAT *ZAG* gene expression. Besides, this subgroup also showed a significant decrease in total cholesterol, triglycerides, and GGT at the end of the study.

### 3.4. Correlation Analysis between Basal SAT and VAT ZAG Gene Expression and BS Outcomes

**Figure 3:**
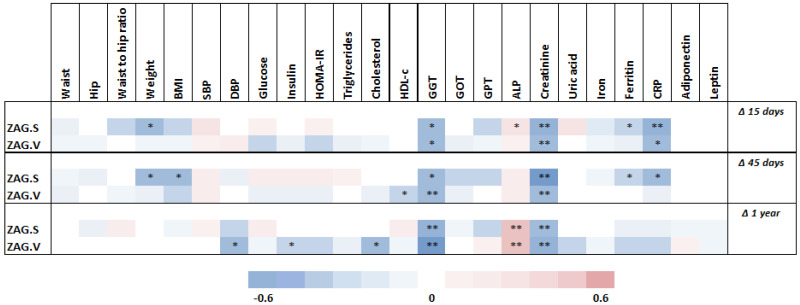
Correlations between clinical and biochemical parameter changes after surgery and basal adipose tissue ZAG levels. Abbreviations: Δ, percentage decrease ((initial value − final value)/initial value)*100; ALP, alkaline phosphatase; BMI, body mass index; CRP, c reactive protein; DBP, diastolic blood pressure; GGT, gamma-glutamyl transferase; GOT, glutamic oxaloacetic transaminase; GPT, glutamic pyruvic transaminase; HDL-c, high-density lipoprotein cholesterol; HOMA-IR, homeostasis model assessment of insulin resistance; SBP, systolic blood pressure; WHR, waist to hip ratio; ZAG.S; subcutaneous adipose tissue ZAG; ZAG.V; visceral adipose tissue ZAG. Statistical significance is expressed as: * *p* < 0.05; ** *p* < 0.01.

A significant negative correlation was found between basal SAT *ZAG* gene expression and weight loss from baseline at 15 and 45 days (*p* < 0.05), whereas no statistically significant correlation was detected with regard to this variable and basal SAT *ZAG* gene expression at 1 year (Figure 3). In addition, basal SAT *ZAG* gene expression also correlated negatively with BMI reduction at 45 days (*p* < 0.05), CRP reduction at 45 days (*p* < 0.05), and GGT decrease at 1 year (*p* < 0.01), among others. On the other hand, a significant inverse correlation was observed between basal VAT *ZAG* gene expression and the decrease in GGT levels from baseline at 1 year (*p* < 0.01), as well as in diastolic blood pressure (*p* < 0.05), insulin (*p* < 0.05), and total cholesterol (*p* < 0.05) at 1 year. 

### 3.5. Variables Associated with Basal SAT and VAT ZAG Gene Expression Levels

Linear regression analyses were performed in order to detect independent associations between basal SAT and VAT *ZAG* gene expression levels and changes in several variables after BS (Table 4 and Table 5). We also analyzed the impact of sex and the presence of metabolic syndrome, as there were differences in ZAG percentile groups. Regression models suggested that basal SAT *ZAG* gene expression levels could explain 14% and 15% of the variation of weight loss and BMI at 45 days after surgery, whereas no associations were observed between basal VAT *ZAG* gene expression levels and weight loss over time. Both basal SAT and VAT *ZAG* gene expression levels could explain between 20–30% of the variation of GGT after BS and between 14–23% of the variation of creatinine after BS. 

## 4. Discussion

In this study, we found that basal SAT and VAT *ZAG* gene expression levels were associated with adverse metabolic features in individuals with class III obesity. Additionally, our results reveal that basal SAT *ZAG* gene expression levels are inversely related to short-term weight loss after BS, whereas no medium-term associations between basal SAT/VAT *ZAG* gene expression levels and weight loss after BS were detected. Several studies have described a close relationship between *ZAG* gene expression levels and obesity, as well as other components of the metabolic syndrome. In this sense, Gong et al. found that serum *ZAG* levels were negatively associated with body weight and body fat percentage [15], and Wang et al. showed that serum ZAG levels were decreased in patients with metabolic syndrome and central obesity [11]. In patients with class III obesity, an inverse relationship between the degree of IR and VAT *ZAG* gene expression levels has been reported, and both VAT and SAT *ZAG* gene expression showed a direct relationship with the genetic expression of lipolytic enzymes [12]. Thus, *ZAG* gene expression has been postulated as a master regulator of lipid metabolism and a key adipokine in the pathophysiological pathways involved in obesity and metabolic syndrome [16]. In line with the above, our results showed an independent inverse relationship between basal SAT *ZAG* gene expression levels and waist circumference and a negative association between basal VAT *ZAG* gene expression levels and waist circumference, weight, BMI, and some components of the metabolic syndrome, such as HOMA-IR, insulin, and triglycerides.

Interestingly, *ZAG* gene expression has been reported to be down-regulated in liver tissues of subjects with non-alcoholic fatty liver disease (NAFLD), and over-expression of *ZAG* may play a protective role against this disease [17,18]. In our population, the lower percentiles of both SAT and VAT *ZAG* gene expression levels presented higher serum levels of GGT as compared to the higher percentiles, and the correlation analysis revealed an inverse relationship between basal SAT *ZAG* gene expression levels and both GGT and GPT. In addition, the correlation analysis and the linear regression showed that basal VAT *ZAG* gene expression levels and GGT were closely associated. These results could be related to a higher prevalence of NAFLD in subjects with decreased *ZAG* gene expression levels, although it is important to bear in mind that the presence of this disease was not specifically assessed in the study, and normal values of GGT and transaminases do not exclude the presence of NAFLD. On the other hand, a positive correlation was observed between SAT/VAT *ZAG* gene expression levels and serum levels of creatinine, and independent associations were also found between these parameters after surgery. In this line, elevated circulating levels of ZAG have been linked to kidney impairment [19]. However, since patients included in this study did not present kidney disease, these results should be cautiously interpreted. 

In addition to its value as a marker of metabolic derangement in patients with obesity, pre-operative *ZAG* gene expression levels might affect BS outcomes. In this regard, several changes in basal adipokine expression and pro-inflammatory biomarkers have been suggested to predict BS outcomes [20]. As an example, higher baseline serum levels of CRP were associated with increased weight loss after BS [21]. On the other hand, it is also important to note that extreme body weight may be related to increased inflammatory parameters, such as CRP, which may be associated with increased morbidity and mortality [22]. Beyond the aforementioned inflammatory markers, the study of several adipokines, such as leptin or adiponectin, as predictors of BS outcomes has led to different results [23,24,25]. However, as far as we know, the role of *ZAG* gene expression levels in predicting BS outcomes has remained unexplored. In the present study, the correlation analysis revealed an inverse relationship between basal SAT *ZAG* gene expression levels and weight loss percentage from baseline at 15 and 45 days after BS, and a close, negative independent relationship was also found between basal SAT *ZAG* gene expression levels and percent weight loss at 45 days after BS. Therefore, as opposed to other basal factors, such as SAT fibrosis, which have been associated with decreased fat mass loss percentage after BS [26], our results show that SAT *ZAG* gene expression levels may have a positive impact on short-term weight loss after BS. Moreover, despite the well-known association between *ZAG* gene expression and several metabolic disorders, such as visceral obesity and metabolic syndrome, decreased basal SAT/VAT *ZAG* gene expression levels were not linked to poorer outcomes in terms of weight loss 1 year after the surgical procedure. 

Notably, both basal SAT and VAT *ZAG* gene expression levels showed a negative association with the decrease of GGT from baseline at 1 year after BS in the linear regression analysis. Since GGT may be a surrogate marker of NAFLD and dynamic changes in serum, GGT levels have been associated with an improvement in liver histology in individuals with NAFLD [27,28], and these results might postulate basal SAT/VAT *ZAG* gene expression levels as a useful marker to predict NAFLD response to BS. However, the patients included in this study did not have a formal diagnosis of NAFLD, and basal GGT levels were significantly higher in the lower percentile of SAT and VAT *ZAG* gene expression levels, which could have led to greater benefits after BS in this population due to altered baseline characteristics. Thus, further research is needed in order to confirm this hypothesis. 

Therefore, in the present study, we show that adipose tissue *ZAG* expression levels are associated to the metabolic status of patients with morbid obesity, and may have an impact on metabolic outcomes related to bariatric surgery. ZAG is a lipolytic adipokine implicated in the regulation of adipose tissue metabolism and distribution, among other functions [29,30]. Therefore, differences in the expression of *ZAG* in adipose tissue may reflect the status of fat metabolism and could explain dynamic changes in adipose tissue after bariatric surgery. Further studies are needed to unravel the exact pathophysiological mechanisms of the reported findings.

The main limitations of our study are the reduced sample sized and its retrospective nature. Therefore, prospective large-scale studies are required to assess the role of *ZAG* gene expression in BS outcomes. Besides, this study was conducted in patients with extreme obesity; hence, these results cannot be extrapolated to other populations. On the other hand, our main strength lies on the novelty of our results as, to our knowledge, this is the first study that evaluates the impact of pre-BS SAT/VAT *ZAG* gene expression levels on short- and medium-term BS outcomes.

In conclusion, lower SAT/VAT *ZAG* gene expression levels were linked to an adverse metabolic profile in patients with class III obesity. Besides, SAT *ZAG* gene expression levels were inversely related to short-term weight loss after BS. Despite the well-described relationship between basal SAT/VAT *ZAG* and obesity severity/metabolic derangement, this feature was not associated with poorer medium-term outcomes (e.g., in terms of weight loss) after BS. Additionally, the decrease of GGT levels showed a negative relationship with basal SAT/VAT *ZAG* gene expression. Further investigation is necessary to confirm the value of basal SAT/VAG *ZAG* gene expression in predicting weight loss and assessing NAFLD improvement after BS. 

## Figures and Tables

**Table 1:** Clinical and biochemical characteristics of studied patients with class III obesity before bariatric surgery (basal) classified by the 50th percentile of SAT and VAT *ZAG* gene expression.

	Subcutaneous ZAG			Visceral ZAG		
	<P50	>P50	P	<P50	>P50	P
N	19	18		20	21	
Gender (F/M)	9/10	15/3	**0.025**	12/8	14/7	0.453
Surgery (S/SG)	16/3	13/5	0.314	17/3	14/7	0.158
Healthy/MS	6/13	7/11	0.452	3/17	11/10	**0.013**
Age (years)	39 ± 10	43 ± 9	0.149	39 ± 10	43 ± 9	0.193
Waist (cm)	146 ± 17	134 ± 12	**0.021**	147 ± 15	134 ± 14	**0.008**
Hip (cm)	158 ± 17	152 ± 12	0.189	158 ± 16	152 ± 13	0.177
Waist to hip ratio	0.92 ± 0.08	0.89 ± 0.07	0.137	0.94 ± 0.10	0.89 ± 0.07	0.074
Weight (kg)	158 ± 31	136 ± 19	**0.012**	154 ± 27	141 ± 25	**0.013**
BMI (kg/m^2^)	56 ± 8	52 ± 6	0.109	56 ± 7	53 ± 8	0.234
SBP (mmHg)	138 ± 15	153 ± 23	**0.029**	147 ± 20	142 ± 22	0.481
DBP (mmHg)	85 ± 13	86 ± 15	0.778	89 ± 15	84 ± 11	0.433
Glucose (mmol/L)	5.7 ± 1.0	5.8 ± 1.1	0.663	6.0 ± 1.2	5.4 ± 0.8	0.176
Insulin (pmol/L)	153 ± 83	153 ± 97	0.685	194 ± 97	111 ± 56	**0.002**
HOMA-IR	5.8 ± 3.8	6.0 ± 4.4	0.916	7.6 ± 4.5	4.0 ± 2.2	**0.003**
Triglycerides (mmol/L)	1.6 ± 0.8	1.4 ± 0.6	0.537	1.7 ± 0.6	1.3 ± 0.7	0.058
Cholesterol (mmol/L)	5.1 ± 1.0	5.0 ± 1.1	0.726	5.2 ± 1.1	4.9 ± 0.9	0.324
HDL-c (mmol/L)	1.1 ± 0.6	1.2 ± 0.2	0.070	1.1 ± 0.2	1.2 ± 0.3	0.068
GGT (U/L)	39 ± 15	31 ± 20	**0.029**	40 ± 21	30 ± 14	0.090
GOT (U/L)	22 ± 7	22 ± 14	0.343	25 ± 14	21 ± 7	0.295
GPT (U/L)	50 ± 18	43 ± 23	0.061	50 ± 24	46 ± 17	0.539
ALP (U/L)	66 ± 24	85 ± 18	**0.012**	73 ± 27	77 ± 21	0.643
Creatinine (μmol/L)	72 ± 13	57 ± 12	**0.004**	68 ± 14	63 ± 15	**0.322**
Uric acid (μmol/L)	399 ± 89	339 ± 89	0.058	393 ± 101	345 ± 71	0.086
Iron (μmol/L)	14 ± 7	16 ± 18	0.707	11 ± 7	18 ± 16	0.032
Ferritin (pmol/L)	258 ± 236	99 ± 106	**0.013**	189 ± 225	155 ± 162	0.572
CRP (mmol/L)	63 ± 50	71 ± 79	0.845	70 ± 45	58 ± 78	0.088
Adiponectin (mg/L)	8.3 ± 3.5	9.8 ± 4.7	0.274	8.1 ± 3.8	9.9 ± 4.1	0.147
Leptin (ng/L)	5.8 ± 2.2	7.0 ± 3.2	0.210	6.1 ± 1.8	6.5 ± 3.3	0.876

Data are presented as means ± SD. Abbreviations: ALP, alkaline phosphatase; BMI, body mass index; CRP, C Reactive Protein; DBP, diastolic blood pressure; F, female; GGT, gamma-glutamyl transferase; GOT, glutamic oxaloacetic transaminase; GPT, glutamic pyruvic transaminase; HDL-c, high-density lipoprotein cholesterol; HOMA-IR, homeostasis model assessment of insulin resistance; M, male; MS, metabolic syndrome; S, Scopinaro technique; SG, sleeve gastrectomy; SBP, systolic blood pressure; ZAG, zinc-α2 glycoprotein. Numbers in bold denote statistically significant differences.

**Table 2:** Changes in clinical and biochemical variables in patients with class III obesity before bariatric surgery (Basal) and 15 days, 45 days, and 1 year after bariatric surgery according to subcutaneous (SAT) *ZAG* gene expression levels. Patients were stratified by the 50th percentile of SAT ZAG gene expression levels.

		Basal	15 Days	45 Days	1 Year
Waist (cm)	<P50	146 ± 17	136 ± 15 ^a^	131 ± 13 ^a,b^	116 ± 10 ^a,b,c^
	>P50	134 ± 12	131 ± 9	123 ± 11 ^a,b^	106 ± 12 ^a,b,c^
Hip (cm)	<P50	158 ± 17	151 ± 16 ^a^	143 ± 13 ^a,b^	123 ± 14 ^a,b,c^
	>P50	152 ± 12	145 ± 9 ^a^	141 ± 10 ^a^	123 ± 13 ^a,b,c^
Waist to hip ratio	<P50	0.92 ± 0.08	0.91 ± 0.08	0.92 ± 0.09	0.95 ± 0.08
	>P50	0.89 ± 0.07	0.91 ± 0.07	0.87 ± 0.07	0.87 ± 0.08
Weight (kg)	<P50	158 ± 31	145 ± 28 ^a^	135 ± 25 ^a,b^	112 ± 24 ^a,b,c^
	>P50	136 ± 19	128 ± 17 ^a^	121 ± 17 ^a,b^	97 ± 20 ^a,b,c^
BMI (Kg/m^2^)	<P50	56 ± 8	51 ± 8 ^a^	48 ± 6 ^a,b^	38 ± 6 ^a,b,c^
	>P50	52 ± 6	50 ± 5 ^a^	47 ± 6 ^a,b^	36 ± 6 ^a,b,c^
SBP (mmHg)	<P50	138 ± 15	120 ± 11 ^a^	127 ± 10	122 ± 16 ^a^
	>P50	153 ± 23	125 ± 14 ^a^	131 ± 16 ^a^	131 ± 19 ^a^
DBP (mmHg)	<P50	85 ± 13	73 ± 12 ^a^	77 ± 11 ^a^	72 ± 11 ^a^
	>P50	86 ± 15	75 ± 13 ^a^	82 ± 12	81 ± 11
Glucose (mmol/L)	<P50	6.7 ± 1.0	5.4 ± 0.5	5.2 ± 0.6	4.9 ± 0.5 ^a,b^
	>P50	5.8 ± 1.1	5.5 ± 0.7	5.1 ± 0.5 ^a,b^	4.8 ± 0.4 ^a,b^
Insulin (pmol/L)	<P50	153 ± 83	97 ± 28 ^a^	83 ± 35 ^a^	63 ± 21 ^a,b,c^
	>P50	153 ± 97	90 ± 49 ^a^	69 ± 28 ^a^	49 ± 14 ^a,b,c^
HOMA-IR	<P50	5.8 ± 3.8	3.7 ± 1.3	2.8 ± 1.1 ^a,b^	2.0 ± 0.8 ^a,b,c^
	>P50	6.0 ± 4.4	3.5 ± 2.1 ^a^	2.3 ± 0.9 ^a,b^	1.5 ± 0.7 ^a,b,c^
Triglycerides (mmol/L)	<P50	1.6 ± 0.8	2.0 ± 0.6 ^a^	1.5 ± 0.4 ^b^	1.3 ± 0.5 ^b,c^
	>P50	1.4 ± 0.6	1.9 ± 0.8	1.4 ± 0.6 ^b^	1.2 ± 0.6 ^b^
Cholesterol (mmol/L)	<P50	5.1 ± 1.0	4.4 ± 0.9 ^a^	3.8 ± 0.7 ^a,b^	4.0 ± 1.0 ^a^
	>P50	5.0 ± 1.1	4.5 ± 1.0	4.0 ± 1.1 ^a^	4.2 ± 1.2
HDL-c (mmol/L)	<P50	1.1 ± 0.2	0.8 ± 0.2 ^a^	0.8 ± 0.2 ^a^	1.2 ± 0.3 ^b,c^
	>P50	1.2 ± 0.2	0.8 ± 0.2 ^a^	1.0 ± 0.3 ^a^	1.2 ± 0.3 ^b,c^
GGT (U/L)	<P50	39 ± 15	102 ± 65 ^a^	36 ± 21 ^b^	23 ± 17 ^a,b,c^
	>P50	31 ± 20	99 ± 46 ^a^	36 ± 15 ^b^	26 ± 13 ^b,c^
GOT (U/L)	<P50	22 ± 7	38 ± 17 ^a^	38 ± 37	20 ± 7 ^b,c^
	>P50	22 ± 14	45 ± 39 ^a^	34 ± 10 ^a^	19 ± 9 ^b,c^
GPT (U/L)	<P50	50 ± 18	83 ± 45 ^a^	72 ± 56	44 ± 13 ^b,c^
	>P50	43 ± 23	103 ± 80 ^a^	61 ± 20 ^a,b^	40 ± 13 ^b,c^
ALP (U/L)	<P50	66 ± 24	78 ± 24	78 ± 23 ^a^	91 ± 30 ^a^
	>P50	85 ± 18	90 ± 21	91 ± 22	88 ± 25
Creatinine (μmol/L)	<P50	72 ± 13	73 ± 21	69 ± 15	65 ± 19
	>P50	57 ± 12	68 ± 15 ^a^	62 ± 13 ^a^	69 ± 12
Uric acid (μmol/L)	<P50	399 ± 89	446 ± 190	321 ± 54 ^a^	291 ± 48 ^a,b,c^
	>P50	339 ±89	309 ± 143	321 ± 167	238 ± 89 ^a^
Iron (μmol/L)	<P50	14 ± 7	8 ± 4 ^a^	9 ± 4	11 ± 4 ^b^
	>P50	16 ±18	10 ± 3	10 ± 4	11 ± 4
Ferritin (pmol/L)	<P50	258 ± 236	537 ± 434 ^a^	366 ± 602 ^b^	187 ± 189 ^b,c^
	>P50	99 ± 106	348 ± 317 ^a^	182 ± 169 ^a^	137 ± 220 ^b,c^
CRP (nmol/L)	<P50	63 ± 50	87 ± 65	17 ± 23 ^a,b^	4 ± 2 ^a,b^
	>P50	71 ± 79	223 ± 245 ^a^	48 ± 59 ^a,b^	7 ± 9 ^a,b^
Adiponectin (mg/L)	<P50	8.3 ± 3.5	7.9 ± 3.3	10.8 ± 6	11.3 ± 5.7
	>P50	9.8 ± 4.7	10 ± 4.7	13.5 ± 7.2 ^a^	14.6 ± 7.9
Leptin (μg/L)	<P50	5.8 ± 2.3	3.2 ± 1.8 ^a^	2.4 ± 1.0 ^a^	1.7 ± 1.1 ^a,b^
	>P50	7.0 ± 3.2	4.6 ± 2.2 ^a^	36 ± 2.2 ^a^	1.8 ± 1.5 ^a,b,c^

Data are expressed as means ± SD. Abbreviations: ALP, alkaline phosphatase; BMI, body mass index; CRP, c reactive protein; DBP, diastolic blood pressure; GGT, gamma-glutamyl transferase; GOT, glutamic oxaloacetic transaminase; GPT, glutamic pyruvic transaminase; HDL-c, high-density lipoprotein cholesterol; HOMA-IR, homeostasis model assessment of insulin resistance; MS, metabolic syndrome; SBP, systolic blood pressure; ZAG, zinc-α2 glycoprotein. ^a^, *p* < 0.05 differences compared with basal levels; ^b^, *p* < 0.05 differences compared with 15 days after surgery and ^c^, significant differences compared with 45 days after surgery.

**Table 3:** Changes in clinical and biochemical variables in patients with class III obesity before bariatric surgery (Basal) and 15 days, 45 days, and 1 year after bariatric surgery according to visceral (VAT) *ZAG* gene expression levels. Patients are stratified by the 50th percentile of VAT *ZAG* gene expression.

		Basal	15 Days	45 Days	1 Year
Waist (cm)	<P50	147 ± 15	138 ± 12 ^a^	131 ± 10 ^a,b^	115 ± 8 ^a,b,c^
	>P50	134 ± 14	130 ± 12	124 ± 14 ^a,b^	107 ± 13 ^a,b,c^
Hip (cm)	<P50	158 ± 16	149 ± 16 ^a^	145 ± 12 ^a^	125 ± 14 ^a,b,c^
	>P50	152 ± 13	146 ± 10 ^a^	139 ± 11 ^a,b^	119 ± 11 ^a,b,c^
Waist to hip ratio	<P50	0.94 ± 0.10	0.93 ± 0.09	0.91 ± 0.08	0.92 ± 0.09
	>P50	0.89 ± 0.07	0.89 ± 0.07	0.89 ± 0.10	0.90 ± 0.10
Weight (kg)	<P50	154 ± 27	143 ± 26 ^a^	133 ± 23 ^a,b^	111 ± 23 ^a,b,c^
	>P50	141 ± 25	130 ± 20 ^a^	123 ± 19 ^a,b^	96 ± 20 ^a,b,c^
BMI (Kg/m^2^)	<P50	56 ± 7	52 ± 7 ^a^	48 ± 6 ^a,b^	39 ± 6 ^a,b,c^
	>P50	53 ± 8	49 ± 6 ^a^	46 ± 6 ^a,b^	35 ± 5 ^a,b,c^
SBP (mmHg)	<P50	147 ± 20	124 ± 15 ^a^	131 ± 12 ^a^	126 ± 22 ^a^
	>P50	142 ± 22	120 ± 12 ^a^	125 ± 15 ^a^	124 ± 15 ^a^
DBP (mmHg)	<P50	89 ± 15	78 ± 13 ^a^	79 ± 13 ^a^	73 ± 14 ^a^
	>P50	84 ± 11	70 ± 11 ^a^	78 ± 11 ^b^	78 ± 9
Glucose (mmol/L)	<P50	6.0 ± 1.2	5.4 ± 0.6	5.2 ± 0.6 ^a^	4.9 ± 0.4 ^a,b^
	>P50	5.4 ± 0.8	5.4 ± 0.7	5.1 ± 0.4 ^b^	4.7 ± 0.4 ^a,b,c^
Insulin (pmol/L)	<P50	194 ± 97	111 ± 42 ^a^	97 ± 35 ^a^	63 ± 21 ^a,b,c^
	>P50	111 ± 56	83 ± 35^a^	63 ± 21 ^a,b^	42 ± 21 ^a,b,c^
HOMA-IR	<P50	7.6 ± 4.5	4.0 ± 1.6^a^	3.1 ± 0.9 ^a,b^	2.0 ± 0.7 ^a,b,c^
	>P50	4 ± 2.2	3.3 ± 1.8	2.1 ± 0.8 ^a,b^	1.4 ± 0.7 ^a,b,c^
Triglycerides (mmol/L)	<P50	1.7 ± 0.6	1.9 ± 0.5	1.6 ± 0.5	1.3 ± 0.4 ^a,b^
	>P50	1.3 ± 0.7	1.9 ± 0.7 ^a^	1.3 ± 0.5^b^	1.1 ± 0.6 ^b^
Cholesterol (mmol/L)	<P50	5.2 ± 1.1	4.4 ± 0.6 ^a^	3.8 ± 0.5 ^a,b^	3.7 ± 0.7 ^a,b^
	>P50	4.9 ± 0.9	4.5 ± 1.1	4.0 ± 1.1 ^a^	4.5 ± 1.3
HDL-c (mmol/L)	<P50	1.1 ± 0.2	0.8 ± 0.1 ^a^	0.8 ± 0.2 ^a^	1.1 ± 0.2 ^b,c^
	>P50	1.2 ± 0.3	0.8 ± 0.2 ^a^	1.0 ± 0.3 ^a,b^	1.3 ± 0.4 ^b,c^
GGT (U/L)	<P50	40 ± 21	110 ± 83 ^a^	37 ± 23 ^b^	23 ± 18 ^a,b,c^
	>P50	30 ± 14	100 ± 46 ^a^	35 ± 15 ^b^	24 ± 11 ^b,c^
GOT (U/L)	<P50	25 ± 14	40 ± 18 ^a^	38 ± 35 ^b^	18 ± 8 ^b,c^
	>P50	21 ± 7	43 ± 35 ^a^	33 ± 13 ^a,b^	20 ± 9 ^b,c^
GPT (U/L)	<P50	50 ± 24	85 ± 41 ^a^	70 ± 53	41 ± 10 ^b,c^
	>P50	46 ± 17	103 ± 77 ^a^	65 ± 25 ^a,b^	42 ± 15 ^b,c^
ALP (U/L)	<P50	73 ± 27	84 ± 29	82 ± 24	96 ± 32 ^a^
	>P50	77 ± 21	85 ± 23	83 ± 25	79 ± 19
Creatinine (μmol/L)	<P50	68 ± 14	67 ± 17	61 ± 12	63 ± 16
	>P50	63 ± 15	74 ± 19 ^a^	68 ± 16	70 ± 15
Uric acid (μmol/L)	<P50	393 ± 101	416 ± 208	363 ± 143	262 ± 65 ^a,b,c^
	>P50	345 ± 71	351 ± 131	297 ± 71 ^a^	280 ± 77 ^a^
Iron (μmol/L)	<P50	14 ± 7	8 ± 4	9 ± 4	11 ± 4 ^b^
	>P50	16 ±18	10 ± 3	10 ± 4	11 ± 4
Ferritin (pmol/L)	<P50	189 ± 225	463 ± 416 ^a^	348 ± 593 ^b^	164 ± 220 ^b,c^
	>P50	155 ± 162	416 ± 344 ^a^	184 ± 153 ^b^	144 ± 173 ^b^
CRP (nmol/L)	<P50	70 ± 45	127 ± 168	24 ± 29 ^a,b^	7 ± 8 ^a,b^
	>P50	58 ± 78	162 ± 191 ^a^	35 ± 56 ^b^	5 ± 5 ^b^
Adiponectin (mg/L)	<P50	8.1 ± 3.8	8.2 ± 4.2	10.5 ± 6.2	9.2 ± 2.3
	>P50	9.9 ± 4.1	9.3 ± 3.8 ^a^	13 ± 6.6	18.1 ± 7.5 ^b^
Leptin (μg/mL)	<P50	6.1 ± 1.8	3.8 ± 2.0 ^a^	2.8 ± 1.3 ^a^	2.0 ± 1.3 ^a,b,c^
	>P50	6.5 ± 3.3	4.0 ± 2.2 ^a^	3.1 ± 2.1 ^a^	0.9 ± 0.5 ^a,b,c^

Data are expressed as means ± SD. Abbreviations: ALP, alkaline phosphatase; BMI, body mass index; CRP, c reactive protein; DBP, diastolic blood pressure; GGT, gamma-glutamyl transferase; GOT, glutamic oxaloacetic transaminase; GPT, glutamic pyruvic transaminase; HDL-c, high-density lipoprotein cholesterol; HOMA-IR, homeostasis model assessment of insulin resistance; MS, metabolic syndrome; SBP, systolic blood pressure; ZAG, zinc-α2 glycoprotein. ^a^, *p* < 0.05 differences compared with basal levels; ^b^, *p* < 0.05 differences compared with 15 days after surgery; and ^c^, significant differences compared with 45 days after surgery.

**Table 4:** Regression analysis predicting clinical and biochemical changes using sex and subcutaneous *ZAG* (ZAG.S) gene expression levels as independent variables.

	R^2^	F	P		Beta	P	
							Δ 15 days
Creatinine	0.192	8.317	0.007	ZAG.S	−0.438	0.007	
GGT	0.229	5.060	0.012	ZAG.S	−0.278	0.087	
				Sex	−0.316	0.053	
Weight	0.133	5.367	0.026	Sex	−0.365	0.026	
							Δ 45 days
Creatinine	0.227	10.291	0.003	ZAG.S	−0.477	0.003	
Ferritin	0.149	6.117	0.018	ZAG.S	−0.386	0.018	
GGT	0.226	10.222	0.003	ZAG.S	−0.475	0.003	
Weight	0.144	5.903	0.020	ZAG.S	−0.380	0.020	
							Δ 1 year
Creatinine	0.135	5.481	0.025	ZAG.S	−0.368	0.025	
GGT	0.334	17.590	0.000	ZAG.S	−0.578	0.000	

Abbreviations: Δ, percentage decrease ((initial value − final value)/initial value) * 100; GGT, gamma-glutamyl transferase; ZAG.S; subcutaneous adipose tissue ZAG.

**Table 5:** Regression analysis predicting clinical and biochemical changes using metabolic syndrome and visceral *ZAG* (ZAG.V) gene expression levels as independent variables.

	R^2^	F	P		Beta	P	
							Δ 15 days
Creatinine	0.196	9.533	0.004	ZAG.V	−0.443	0.004	
GGT	0.243	12.542	0.001	ZAG.V	−0.493	0.001	
							Δ 45 days
Creatinine	0.141	6.404	0.016	ZAG.V	−0.376	0.016	
GGT	0.206	10.096	0.003	ZAG.V	−0.453	0.003	
							Δ 1 year
ALP	0.112	4.937	0.032	ZAG.V	0.335	0.032	
Creatinine	0.188	9.048	0.005	ZAG.V	−0.434	0.005	
GGT	0.290	15.933	0.000	ZAG.V	−0.539	0.000	
Insulin	0.138	6.236	0.017	ZAG.V	−0.371	0.017	

Abbreviations: Δ, percentage decrease ((initial value − final value)/initial value) × 100; GGT, gamma-glutamyl transferase; ZAG.V; visceral adipose tissue ZAG.

## Data Availability

The data presented in this study are available on request from the corresponding author. The data are not publicly available due to ethical reasons.

## References

[1] Bartolomucci A., Parmigiani S., Rodgers R.J., Vidal-Puig A., Allan S.E., Siegel V. (2012). The Obese Species: A special issue on obesity and metabolic disorders. DMM Dis. Model Mech..

[2] Bray G.A., Heisel W.E., Afshin A., Jensen M.D., Dietz W.H., Long M., Kushner R.F., Daniels S.R., Wadden T.A., Tsai A.G. (2018). The science of obesity management: An endocrine society scientific statement. Endocrinol. Rev..

[3] Ng M., Fleming T., Robinson M., Thomson B., Graetz N., Margono C., Mullany E.C., Biryukov S., Abbafati C., Abera S.F. (2014). Global, regional, and national prevalence of overweight and obesity in children and adults during 1980-2013: A systematic analysis for the Global Burden of Disease Study 2013. Lancet.

[4] Nguyen N.T., Varela J.E. (2017). Bariatric surgery for obesity and metabolic disorders: State of the art. Nat. Rev. Gastroenterol. Hepatol..

[5] Choe S.S., Huh J.Y., Hwang I.J., Kim J.I., Kim J.B. (2016). Adipose Tissue Remodeling: Its Role in Energy Metabolism and Metabolic Disorders. Front. Endocrinol..

[6] Drolet R., Richard C., Sniderman A.D., Mailloux J., Fortier M., Huot C., Rhéaume C., Tchernof A. (2008). Hypertrophy and hyperplasia of abdominal adipose tissues in wome. Int. J. Obes..

[7] Bing C., Mracek T., Gao D., Trayhurn P. (2010). Zinc-α2-glycoprotein: An adipokine modulator of body fat mass. Int. J. Obes..

[8] Mracek T., Ding Q., Tzanavari T., Kos K., Pinkney J., Wilding J., Trayhurn P., Bing C. (2010). The adipokine zinc-α2-glycoprotein (ZAG) is downregulated with fat mass expansion in obesity. Clin. Endocrinol..

[9] Hocking S., Samocha-Bonet D., Milner K.-L., Greenfield J.R., Chisholm D.J. (2013). Adiposity and Insulin Resistance in Humans: The Role of the Different Tissue and Cellular Lipid Depots. Endocrinol. Rev..

[10] Balaz M., Vician M., Janakova Z., Kurdiova T., Surova M., Imrich R., Majercikova Z., Penesova M., Vlcek A., Kiss A. (2014). Subcutaneous adipose tissue zinc-α2-glycoprotein is associated with adipose tissue and whole-body insulin sensitivity. Obesity.

[11] Wang L., Liu M., Ning D., Zhu H., Shan G., Wang D., Ping B., Yu Y., Yang H., Yan K. (2020). Low Serum ZAG Levels Correlate With Determinants of the Metabolic Syndrome in Chinese Subjects. Front. Endocrinol..

[12] Garrido-Sánchez L., García-Fuentes E., Fernández-García D., Escoté X., Alcaide J., Perez-Martinez P., Vendrell J., Tinahones F.J. (2012). Zinc-alpha 2-glycoprotein gene expression in adipose tissue is related with insulin resistance and lipolytic genes in morbidly obese patients. PLoS ONE.

[13] Morse K.M., Astbury N.M., Walczyszyn A., Hashim S.A., Geliebter A. (2017). Changes in zinc-α2-glycoprotein (ZAG) plasma concentrations pre and post Roux-En-Y gastric bypass surgery (RYGB) or a very low calorie (VLCD) diet in clinically severe obese patients: Preliminary Study. Integr. Obes. Diabetes.

[14] Ergun S., Ergun D.D., Akinci O., Taskin H.E., Simsek G., Taskin M., Uzun H. (2022). The role of laparoscopic sleeve gastrectomy on inflammatory parameters in morbidly obese patients. J. Visc. Surg..

[15] Gong F.-Y., Zhang S.-J., Deng J.-Y., Zhu H.-J., Pan H., Li N.-S., Shi Y.-F. (2009). Zinc-α2-glycoprotein is involved in regulation of body weight through inhibition of lipogenic enzymes in adipose tissue. Int. J. Obes..

[16] Banaszak M., Górna I., Przysławski J. (2021). Zinc and the innovative zinc-α2-glycoprotein adipokine play an important role in lipid metabolism: A critical review. Nutrients.

[17] Liu T., Luo X., Li Z.-H., Wu J.-C., Luo S.-Z., Xu M.-Y. (2019). Zinc-α2-glycoprotein 1 attenuates non-alcoholic fatty liver disease by negatively regulating tumour necrosis factor-α. World J. Gastroenterol..

[18] Xiao X.-H., Wang Y.-D., Qi X.-Y., Wang Y.-Y., Li J.-Y., Li H., Zhang P.-Y., Liao H.-L., Li M.-H., Liao Z.-Z. (2018). Zinc alpha2 glycoprotein protects against obesity-induced hepatic steatosis. Int. J. Obes..

[19] Sörensen-Zender I., Beneke J., Schmidt B.M.W., Menne J., Haller H., Schmitt R. (2013). Zinc-alpha2-glycoprotein in patients with acute and chronic kidney disease. BMC Nephrol..

[20] Keshavjee S.H., Schwenger K.J.P., Yadav J., Jackson T.D., Okrainec A., Allard J.P. (2021). Factors Affecting Metabolic Outcomes Post Bariatric Surgery: Role of Adipose Tissue. J. Clin. Med..

[21] O’Rourke R.W., Johnson G.S., Purnell J.Q., Courcoulas A.P., Dakin G.F., Garcia L., Hinojosa M., Mitchell J.E., Pomp A., Pories W.J. (2019). Serum biomarkers of inflammation and adiposity in the LABS cohort: Associations with metabolic disease and surgical outcomes. Int. J. Obes..

[22] Duchnowski P., Hryniewiecki T., Kusmierczyk M., Szymanski P. (2018). The usefulness of selected biomarkers in patients with valve disease. Biomark. Med..

[23] Carbone F., Migliola E.N., Bonaventura A., Vecchié A., De Vuono S., Ricci M.A., Vaudo G., Boni M., Dallegri F., Montecucco F. (2018). High serum levels of C-reactive protein (CRP) predict beneficial decrease of visceral fat in obese females after sleeve gastrectomy. Nutr. Metab. Cardiovasc. Dis..

[24] Czupryniak L., Pawlowski M., Kumor A., Szymanski D., Loba J., Strzelczyk J. (2007). Predicting Maximum Roux-en-Y Gastric Bypass-Induced Weight Reduction—Preoperative Plasma Leptin or Body Weight?. Obes. Surg..

[25] Herder C., Peltonen M., Svensson P.-A., Carstensen M., Jacobson P., Roden M., Carlsson L., Sjöström L. (2014). Adiponectin and Bariatric Surgery: Associations With Diabetes and Cardiovascular Disease in the Swedish Obese Subjects Study. Diabetes Care.

[26] Divoux A., Tordjman J., Lacasa D., Veyrie N., Hugol D., Aissat A., Guerro-Milo M., Poitou C., Zucker J.-L., Clément K. (2010). Fibrosis in Human Adipose Tissue: Composition, Distribution, and Link With Lipid Metabolism and Fat Mass Loss. Diabetes.

[27] Dixon J., Bhathal P., O’Brien P. (2006). Weight Loss and Non-alcoholic Fatty Liver Disease: Falls In Gamma-Glutamyl Transferase Concentrations are Associated with Histologic Improvement. Obes. Surg..

[28] Newton K.P., Lavine J.E., Wilson L., Behling C., Vos M.B., Molleston J.P., Rosenthal P., Miloh T., Fishbein M.H., Jain A.K. (2021). Alanine Aminotransferase and Gamma-Glutamyl Transpeptidase Predict Histologic Improvement in Pediatric Nonalcoholic Steatohepatitis. Hepatology.

[29] Escoté X., Aranda G., Mora M., Casals G., Enseñat J., Vidal O., Esteban Y., Halperin I., Hanzu F.A. (2017). Zinc alpha-2 glycoprotein is overproduced in Cushing’s syndrome. Endocrinol. Diabetes Nutr..

[30] Hassan I., Waheed A., Yadav S., Singh T.P., Ahmad F. (2008). Zinc A2-Glycoprotein: A Multidisciplinary Protein Md. Mol. Cancer Res..

